# Challenges and Future Perspectives for Additively Manufactured Polylactic Acid Using Fused Filament Fabrication in Dentistry

**DOI:** 10.3390/jfb14070334

**Published:** 2023-06-22

**Authors:** Ghais Kharmanda

**Affiliations:** 1 Mechanics Laboratory of Normandy, INSA Rouen, 76800 St Etienne du Rouvray, France; 2 3D printing 4U (UG), Nördlinger Str. 10, 51103 Cologne, Germany; ghais.kharmanda@3d-printing-4u.com

**Keywords:** additive manufacturing, fused filament fabrication, polylactic acid, dentistry

## Abstract

Additive manufacturing (AM), which is also called rapid prototyping/3D printing/layered manufacturing, can be considered as a rapid conversion between digital and physical models. One of the most used materials in AM is polylactic acid (PLA), which has advantageous material properties such as biocompatibility, biodegradability, and nontoxicity. For many medical applications, it is considered as a leading biomaterial. In dentistry, in addition to its uses in dental models (education, teaching, simulation needs), it can be used for therapeutic objectives and tissue engineering. The fused filament fabrication (FFF) technique, also called fused deposition modeling (FDM), is widely used as an AM technique to perform complex and functional geometries directly from CAD files. In this review, the objective was to present the different challenges and future perspectives of this additively manufactured material by using FFF in dentistry areas. Some suggestions for future directions to extend to more dental applications (support structures, lattice structures, etc.) and to consider more criteria (sustainability, uncertainty etc.) will be discussed. Advanced studies such as machine learning (ML) techniques will be suggested to reduce the failure cases when using the additively manufactured PLA by FFF in dentistry.

## 1. Introduction

**Figure 1:**
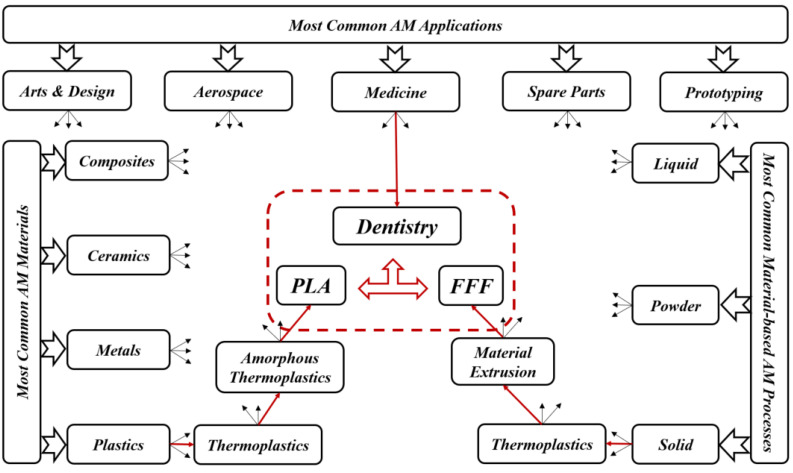
Roadmap of current review topics.

Traditional methods of transplantation of organs have several risks such as limited sources, complications, and secondary injuries. Additive manufacturing (AM) technology potentially helps to solve these issues since it can rapidly fabricate personalized tissue structures such as scaffolds, repair certain tissue defects, and even directly manufacture tissues and organs [1]. It is true that these types of additively manufactured structures cannot perfectly match the patient’s damaged tissue, but they may possess suitable microstructures and cell arrangements for the promotion of cell growth and differentiation. In this review, we deal with the dental applications where additively manufactured PLA (AM-PLA) using fused filament fabrication (FFF) technique is used. For selecting the topic of this review, Figure 1 presents the roadmap of the three main axes. The first axis is represented by the most common AM applications that can be found in the following areas: aerospace, spare parts, arts and design, prototyping, and medicine. The medical applications can be divided into orthopedics and orthodontics. Using AM technology, it is possible to build complicated models for surgery preparation. AM models of a patient’s anatomy can be very helpful to better understand the patient’s anatomy prior to surgery instead of using CT scans and MRI. In addition, these models can be used for surgical simulation and training purposes [2,3,4]. Dentistry involves the diagnosis, treatment, and condition counteraction, turmoil, and sicknesses of the teeth, gums, jaws, and mouth. It is frequently viewed as fundamental for complete oral well-being. The human mouth’s oral cavity includes the upper and lower jaws, which are respectively called maxilla and mandible. Each jaw (upper/lower) contains 16 teeth, nerves, blood vessels and muscles [5]. Dentistry was selected to be treated in this review since it can affect the whole human body’s health. The second axis is represented by the most common AM materials: plastics, metals, ceramics, and composites [6]. Plastic materials have two types: thermosets and thermoplastics. Thermoplastic materials are utilized in two kinds of AM techniques: powder bed fusion and material extrusion. Among these kinds, amorphous thermoplastic materials are utilized for material extrusion processes due to their melt properties (high viscous melt). The typical size of the nozzle used for extrusion of these materials is 0.2–0.5 mm [7]. Polylactic acid (PLA) [8,9] and acrylonitrile butadiene styrene (ABS) [10,11] are the two most common examples. Polycarbonate (PC) [12], PC/ABS blend [13], and polyetherimide (PEI) [14] are other examples of amorphous materials that are used in material extrusion. In this review, we focus on the PLA material due to its advantageous properties. The thirst axis is represented by the most common material-based AM processes. The classifications of these processes are based on the material states: solid, liquid, and powder. They can be respectively labeled as follows: solid-based AM methods, liquid-based AM methods, and powder-based AM methods [15]. Material extrusion, powder bed fusion directed energy deposition material jetting, binder jetting, vat photopolymerization, and sheet lamination are among the seven additive manufacturing techniques classified by ASTM/F2921 [4]. Extrusion-based techniques, being cost-effective, are widely used for solid materials. Several materials such as multi-colored plastics and living cells are manufactured following a material extrusion-based technology [16]. Additionally, this process can be completely based on the functional aspects of the products [17,18]. Among these techniques, fused filament fabrication (FFF), also called fused deposition modeling (FDM), is a desirable AM technique in order to fabricate PLA due to its geometrical flexibility and relatively low cost. An illustration and details of the FFF technique can be found in our previous work [19]. Using the FFF technique, several dental applications can be performed in a simple way. For example, with this technique, it is possible to produce polymer dentures with hollow, semi-hollow, and solid structures [20].

Several AM techniques used in the dentistry field are first presented in this review to show their different uses for dental applications. Next, we present the different AM materials used in dentistry. After that, we focus on AM-PLA (properties, advantages, etc.) and its applications to dentistry. The different advantages and disadvantages of the used AM technique (FFF) and its dental applications will be discussed later. Thereafter, three sections are presented to consider sustainability, uncertainty, and artificial intelligence for this topic. Before the conclusion, we present the different challenges, issues, and some future perspectives to extend the use of AM-PLA by FFF to several dental applications. 

## 2. AM Techniques Used in Dentistry

Day by day, additive manufacturing technology is expanding, and, because of its frequently used layer-by-layer construction, it has a remarkable perspective for biomedical applications. This technology may directly fabricate specific functional components with the help of scanned data in CT images that give superior visualization of a particular framework. In typical presurgical preparation, it helps medical experts to practically replan surgical events. In addition, it can be considered as a communication tool between medical experts and patients [15,21].

**Figure 2:**
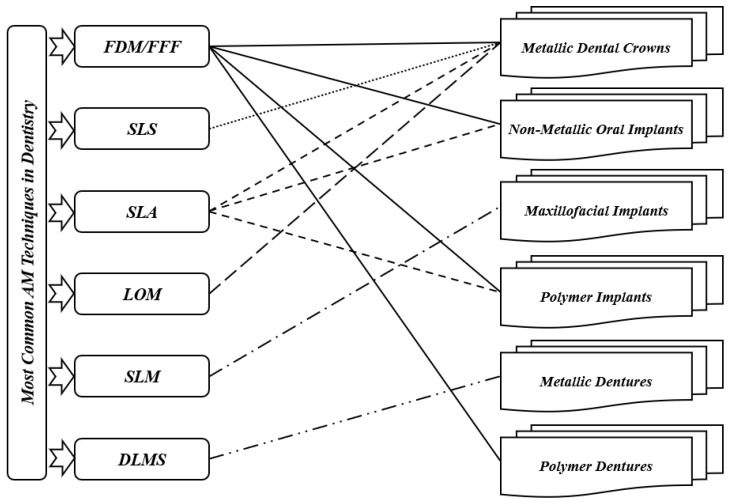
Most common AM techniques in dentistry.

Fields such as dentistry, where the anisotropy is needed as per the requirement, pose as best clients for AM technology. Implant section is one of the most important medical applications for AM. The implants are customized as per the needs and patient’s requirements; AM can then help in fabricating those implants as required [22]. For dental applications, models, splints, and drill guides are developed by AM technology. In addition, the development of artificial tissues and organs has been carried out by using AM techniques [23]. AM techniques are currently widely used for 3D organ models, which are useful to understand complex human anatomy. In a recent review by Rouf et al. [6], several AM techniques for various aspects of medical needs (orthopedics and orthodontics) were presented. In this section, some AM works in dentistry are presented since AM for dentistry has been applied for almost 20 years. As shown in Figure 2, for metallic dental crowns, researchers used several AM techniques such as fused filament fabrication (FFF) [24], selective laser sintering (SLS) [25], stereolithography (SLA) [26], and laminated object manufacturing (LOM) [27]. Dental pieces, bridges and crowns were the dental applications for these AM techniques [28,29]. For non-metallic oral implants, crowns and bridges, models for dental study, and surgical equipment (specifically surgical guides for dental surgery), SLA and FFF are generally used [30]. For maxillofacial implants, the metallic powder using the selective laser melting method (SLM) [31] replaces the patient’s entire jaw. Furthermore, AM technologies have been used for creating complete or partial dentures, where direct laser metal sintering (DLMS) processes have been utilized to create metallic dentures [32,33,34]. The FFF technique has been used to create polymer dentures with solid, hollow, or semi-hollow structures [20]. Research is currently being conducted using AM techniques to develop dentures that possess anti-microbial properties [35]. FFF and SLA have been used to generate bioresorbable polymer dental implants that display odontogenic properties [36,37]. Recently, powder bed fusion technologies became leading AM techniques in dental applications [6]. However, the roughness associated with printed components can represent a real issue. So, there is a need to remedy the roughness in dental implants. On the other hand, FFF technique can be utilized in order to create complex and functional geometries, starting directly from CAD models [38]. This technique can then be employed in order to produce cellular structures with controllable pore shape, pore size, and porosity. These kinds of structures are fundamental in orthopedic scaffolds due to their high compressive strength, low elastic modulus, and adequate cell accommodation spaces. Several developments are needed to extend the application of these advantageous structures to dentistry, especially when a patient suffers from bone loss due to injuries or accidents. Furthermore, other AM applications can be found in maxillofacial surgery. For example, it is possible to develop surgical equipment to correct facial defects. This kind of equipment can reduce the surgery risks and provide aesthetic results [39]. By developing 3D inkjet-printed bones in the jaw area, a group of researchers found a solution for mandibular deformities [40]. These fabricated bones had dimensional compatibility in patients, and there was a link between artificial bones and host ones. That can allow reducing operational time and risk and simplify adjusting the size for bone fixation during treatment. In addition to these applications, splints can be performed by using AM techniques. Sun et al. [41] provided AM splints for maxillofacial surgery that helped to identify the positions of mandible and maxilla as parts of facial bone treatment. These splints showed high accuracy levels and improved mechanical strength. For mandible fractures or defects, AM technology also helped to provide bone implants. As part of surgical treatment for square-shaped or asymmetric faces, titanium implants based on the anatomy of the patient’s mandible have been developed, and part of the mandible has been replaced with that. With this implant invention, it is much easier for surgeons to treat mandible defects and fractures that were very difficult to solve in the early stages [42]. When dealing with neurosurgery, additively manufactured skull models are also useful for skull defects. In the next sections, FFF is selected to be treated among the different AM techniques due to its various advantages. 

## 3. AM Materials Used in Dentistry

Ceramics, metals, and polymers are the most common materials utilized for dental applications such as crowns, bridges, implants, splints, etc. It is true that ceramics and metals are generally preferred for many dental applications; however, polymers are used for biodegradable applications [5]. In literature, many materials have been used in dentistry. However, in this section, we present only some recent studies of AM materials applied to dentistry. Bae et al. [43] used SLM and cold isostatic pressing (CIP) techniques for 3Y-TZP ceramics for fabrication of dental crowns and prostheses and for dental restoration. According to their study, the maximum flexural strength and maximum densification were achieved through sintering at 1500 °C. Their method led to the establishment of SLS/CIP technology for 3Y-TZP ceramics. Next, Muta et al. [44] used the FFF technique for polyvinyl alcohol (PVA) material to fabricate provisional dental crowns. They found that with good accuracy, additively manufactured PVA models can be utilized to fabricate crowns. In the same period, a research paper on the use of alumina ceramics with the FFF technique to fabricate dental crowns was published by Arnesano et al. [45]. At 1150 °C, it was found that the used samples were pre-sintered, and the mechanical properties were like those of pure alumina. Their method was cost-effective and energy-efficient. Revilla-León et al. [46] used SLM and conventional milling (CM) techniques with cobalt chromium alloy (Co-Cr) to fabricate dental prostheses. When fabricating the samples by the two processes, it was found that the shear bond strength had no significant impact, while the roughness was enhanced with the SLM process. In the same period, Baciu et al. [47] published a research paper on using Co–Cr–W alloy with the SLM technique to fabricate dental bridges and inlays. A modification in roughness was found after fabricating specimens using different blasting media. For more information about the different materials used in dentistry, such as titanium and other alloys, the interested reader can refer to [5,6]. Table 1 presents a summary of the different results of recent material studies in dentistry. According to this table, there is no recent work studying AM-PLA for dental applications, while one can find many recent works that aimed to improve the mechanical properties of AM-PLA parts in different areas [19]. The researchers focused on studying the mechanical properties of AM-PLA parts, considering simple specimens (simple geometrical models); however, when dealing with dental applications, complex geometrical models that can largely affect the mechanical properties of the final printed parts should be considered.

In general, and according to our best knowledge, there have been no significant developments that use PLA materials in dental applications during the last three decades despite this material possessing many advantageous properties. In the next sections, we only focus on PLA material for dentistry in order to extend its applications considering several criteria such as sustainability, biodegradability, and biocompatibility. To meet this objective, some suggestions will be added, especially when dealing with composite PLA materials to improve their mechanical properties. 

## 4. AM-PLA Material and Its Application to Dentistry

It is known that one of the most utilized AM materials is PLA since it is considered as a biocompatible, biodegradable, and nontoxic material [48,49,50,51]. Several works have been carried out to identify the design and process parameter effects on the quality of the final product [52]. PLA material is considered as a leading biomaterial for many medical applications and may replace conventional petrochemical-based polymers [53,54]. Due to its high potential for applicability in several areas such as medicine, chemistry, and biotechnology, it has been considered as a promising product under the concept of “green plastic”, since most of the polymers currently produced, are petroleum-based and non-renewable raw materials. The availability of pure lactic acid isomers is considered as an essential aspect for producing PLA with more interesting thermal and mechanical properties. In addition to its low environmental effects, it can be recycled in a traditional way [55]. This enhances its use as a promising polymer in medical applications, especially dental ones. Ramot et al. [56] reviewed the inflammatory reaction process that can be expected following PLA implantation, and they highlighted specific cases in which the inflammatory reaction could lead to some safety issues. In addition, some cases from different medical fields have been reported with the objective of demonstrating possible clinical side effects due to its use. Two kinds of biomaterials can be primarily utilized to prepare biodegradable scaffolds and medical models: natural and synthetic biomaterials. Usually, chitosan, fibrin, and collagen are utilized as natural medical polymeric materials that have excellent compatibility levels, stimulate cell adhesion and proliferation, and also maintain cell phenotypes; however, they can lead to poor mechanical strength (can be easily deformed). The degradation time, shape, and relative molecular mass of synthetic polymers such as PLA, PVA, and polycaprolactone (PCL) [57] can be precisely controlled. However, the polymer surfaces lack recognition sites for cell adhesion, which leads to heterogeneous cell distribution and then cell loss. Therefore, the mechanical performance of the polymer, such as fluidity and surface roughness, must be enhanced for it to be used in medical implants. Yue et al. [58] integrated more complex functions into polymers. After preparing antimicrobial composite resins, they found that antibacterial printed implants killed bacteria on contact without damaging human cells and may be eventually used to replace conventional dental fillings. Furthermore, their approach utilized for fabricating antimicrobial polymers can easily be transferred to other, nonmedical applications such as food packaging, water purification, or even toys for children. Despite PLA material having many advantages, it is difficult for the moment to use it in permanent replacements such as inlays and onlays since it may lead to total or partial loss of these replacements. So, for inlays and onlays, it is recommended to continue using ceramic/porcelain, gold, resin, zirconia materials (alloys), and metals (alloys) such as gold, palladium, chromium, nickel, etc., for permanent crowns and bridges. According to our best knowledge, dental PLA models such as replacement teeth are largely used for simulation needs (teaching, training, etc. This material can also be used in plates and tissues [1] for treating maxilla and mandible fractures. In certain periodical (provisional) parts (crowns, bridges, plates, etc.) this material can be used during the treatment period because of its biodegradability properties. 

## 5. The FFF Technique and Its Application to Dentistry

**Figure 3:**
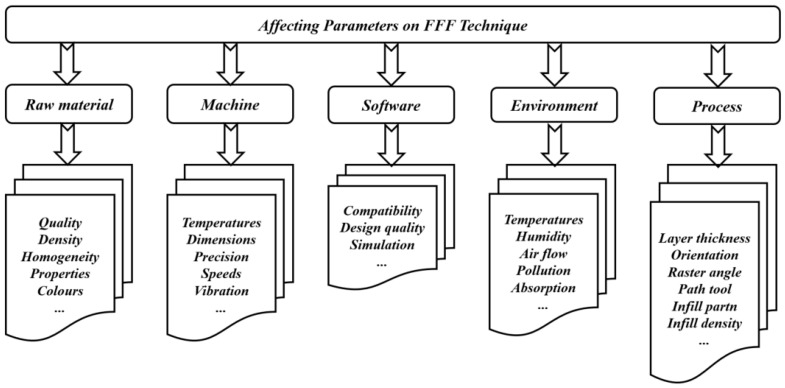
Impacts of different parameters when using FFF technique.

One of the most common material extrusion manufacturing methods is the FFF technique [24], where the filament is a thermoplastic type considered as the input material. The filament is fed into a heated nozzle, where it is melted and squeezed out to form layers. The thickness and pattern of layers of a desired design should be provided to slicing software. After completing one layer, the build platform lowers down automatically, and the printing of the second layer starts. The AM process continues until obtaining the final product. In some machines, two nozzles can be used: one for the desired printed part and the other for support structure material. The nozzle is heated in order to melt the filament and moves accordingly to produce layers to print the desired part. Although the FFF technique provides good accuracy and low maintenance costs, seam line formation problems may occur [15,59]. In addition, in order to improve the accuracy and the quality of the final products, other defects such as voids and weak bonding between printed layers should be considered as common types of defects to be treated in the FFF process for different dental applications [20]. However, the important feature of the FFF technique is its ability to fabricate products with functionally graded properties [60]. Recent works have developed this technique in such a way that it is directly being utilized to fabricate products rather than prototypes. Several parameters affecting the properties of products manufactured using this technique are shown in Figure 3. These parameters are classified according to the whole printing cycle, starting from raw materials to the environmental stages. Recently, several works have been conducted to test parameters of the FFF techniques. For example, the effects of various printing parameters, such as nozzle temperature [61], layer thickness [62], raster angle [61,63], and specimen orientation [64,65,66], have been studied, along with their influence on several structural, mechanical, and tribological properties. Secondary parameters, such as environmental parameters, have also shown their influence on the mechanical properties of materials manufactured with the FFF technique [67,68,69]. From the literature, the effectiveness of the FFF technique has been shown in terms of porosity, while in the case of mechanical properties, the SLS technique is much more dominant [6]. In addition, the process parameters have an important role in deciding the mechanical and tribological properties of the additively manufactured products. So, the influence of each printing parameter on the properties should be understood and considered independently.

In this review, we deal with the advantages and possible developments of the FFF technique for AM-PLA material to extend its applications in dentistry. For example, in the works by Myers et al. [38], the objective was to optimize the FFF process parameters for PLA material considering two lattice structures: Schoen Gyroid and Schwarz Primitive models. They analyzed the impact of the critical process parameters such as flow rate, print speed, and layer height on the geometrical accuracy and compressive strength. This idea was largely applied to orthopedical applications, while there is a need to extend the application of PLA materials (pure and composite) using the FFF technique to orthodontic areas, taking biodegradability and biocompatibility into account.

## 6. AM-PLA Using FFF for Dentistry with Considerations for Sustainability

**Figure 4:**
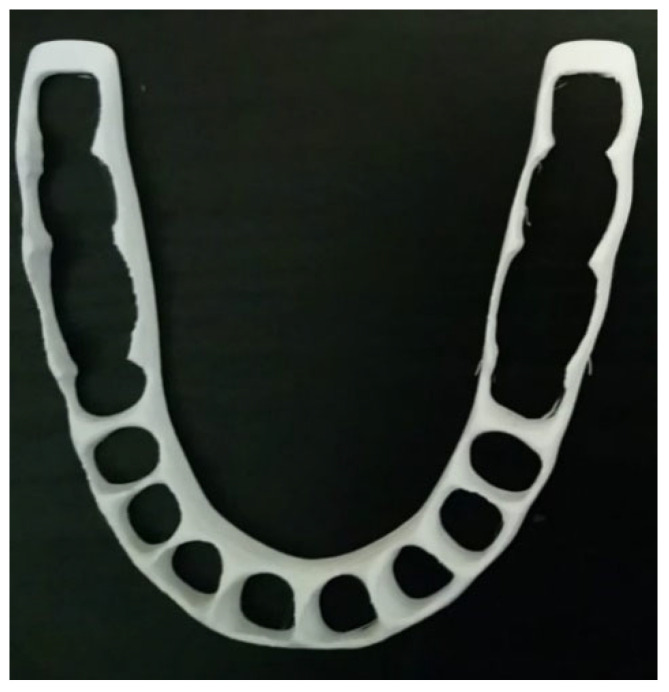
Cap splint (image belongs to 3d-printing-4u.com).

The accelerated level of manufacturing growth cannot always optimize natural resource consumption; it may lead to the waste of materials and costs, which has a big influence on the environment [70,71]. Therefore, many efforts are needed to integrate sustainability into manufacturing. In general, sustainability can be represented by three dimensions: economic, social, and environmental. These dimensions are addressed according to the 6R Concept: Reduce/Recover/Recycle/Reuse/Redesign/Remanufacture [72]. Implementing AM technologies simplifies the traditional supply chain and significantly reduces costs related to transport and warehousing. Furthermore, it allows for considerably reduced generation of waste. During the last decade, more attention to the sustainability of additive manufacturing has started to be applied [70]. Despite these advantages of AM technology, the high price of machinery and the lack of current knowledge prevent it from spreading widely [73]. According to a recent review by Hegab et al. [74], it was shown that AM provides sustainability advantages at various phases of the product life cycle. While AM may be regarded as a direct replacement for conventional manufacturing techniques, its greatest economic advantages are seen in the manufacture of customized single or small batches of items. Sustainability is a very essential objective to be considered in the progress of this field. Here, the objective is to focus on sustainability when developing new PLA-based alloys. Biocompatibility and biodegradability should also be considered during the alloy preparation. When performing the tissue implementation, many complications can be avoided or reduced. In addition, for the FFF technique, few works have been carried out to optimize the support structure and printing path for material reduction [75]. Despite AM being material-efficient [76,77], its efficiency has several issues in practice due to its low productivity. To pave the way for solving this problem, we selected a simple AM technique such as FFF to deal with several issues related to it. For example, when dealing with splint structures, the use of PLA-based materials by the FFF technique is an important subject to develop. Figure 4 shows a simplified model of a printed cap splint (PLA using FFF), which can be used as a fixation technique during the healing period of mandibular fracture cases. The cap splint technique is one of the used mandibular fracture fixation techniques [78]. The shape of a splint can be easily modeled and additively manufactured (using FFF) for a specific patient and leads to a reduction in complications (reduced trauma, especially in the surrounding tissues). In addition to their biocompatibility advantages during the healing period, the removed splints can be recycled after use. 

**Figure 5:**
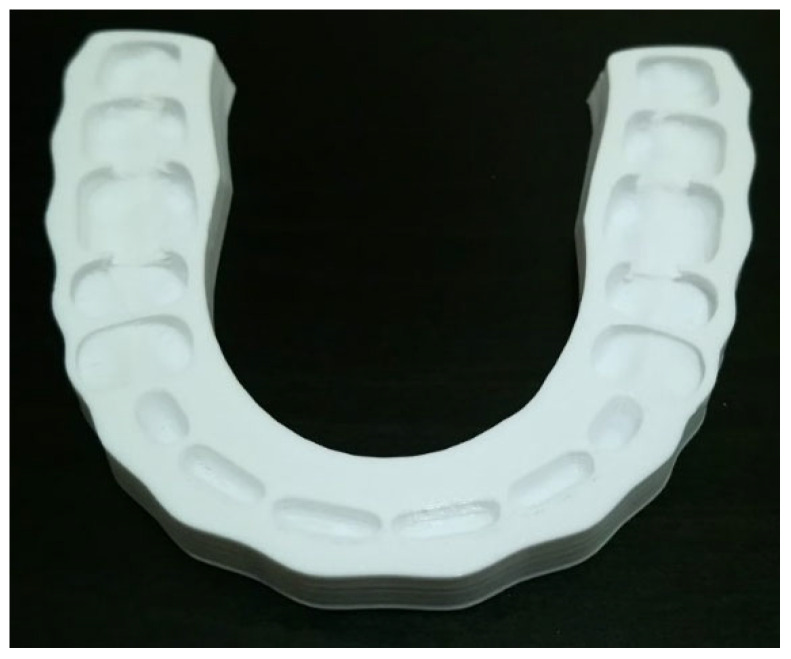
Occlusal splint (image belongs to 3d-printing-4u.com).

Another example, illustrated in Figure 5, is an occlusal splint (dental, night or bite guard). For more than a hundred years, occlusal splints have been utilized to deal with jaw dysfunction. Several kinds of occlusal splints can be found in the market, such as stabilization and palatal splints [79]. They can be used to protect teeth, jaws and muscles from wear, damage etc. This kind of splint is recommended for use when sleeping and even during the day if necessary to deal with problems with stress or anxiety. Currently, these occlusal splints are made of resin alloys; however, to use AM-PLA materials with FFF, several developments are needed to improve their mechanical properties, such as elasticity and adhesion. Furthermore, occlusal harmony between the remaining natural teeth and a removable partial denture plays an important role in preserving the health of the surrounding structure [80]. The biocompatibility of PLA material and the improvement of its mechanical properties may lead to harmonious occlusion during the patient’s treatment. 

These applications and developments largely help to increase the use of PLA from a sustainability point of view (both economically and environmentally). In dentistry, two research axes are needed: the first one is to find more applications for this sustainable material, while the second is to develop new strategies to improve its mechanical properties by optimizing printing parameters and/or mixing it with additional sustainable materials (composite PLA materials).

## 7. AM-PLA Using FFF for Dentistry with Consideration for Uncertainty

**Figure 6:**
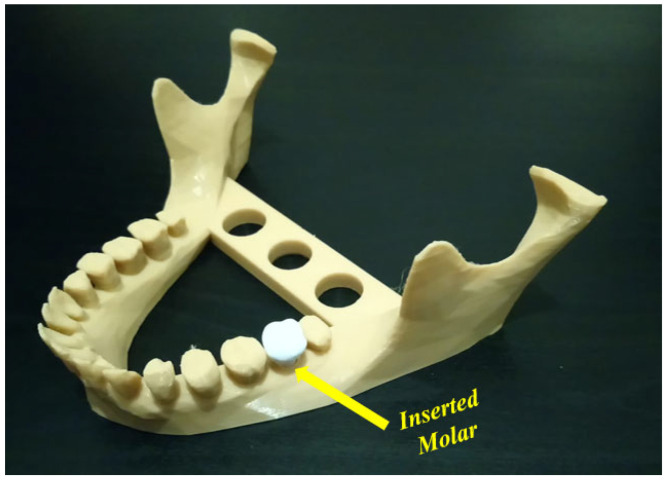
Inserted molar in a mandible model (image belongs to 3d-printing-4u.com).

Uncertainty analysis seeks to study how the variation in the probabilistic output of a model can be apportioned to different sources [81]. To apply the uncertainty concept to additively manufactured PLA using the FFF technique, effective parameters at the different stages should be first identified [19,82]. Uncertainty can be found in the design and process stages [83] and leads to several risks. When applying the FFF technique to polymer materials, the resulting printed parts are still currently used as conceptual prototype components. These printed components are not considered as functional elements since they possess poor mechanical properties [84]. In this way, several sources of uncertainty may appear, and it is important to evaluate the different risks resulting from these uncertainties. At the design stage, the appearance of uncertainty in geometric models may lead to several manufacturing issues where some deviations from the as-designed shape can be shown [85]. In addition to geometric uncertainty, uncertainty can appear in the material properties. These properties are affected by several parameters such as extrusion, direction, and orientation, which impact the manufacturing cost [86,87]. Furthermore, the increased likelihood of failure due to uncertainty problems represents an important point to consider. It can be confusing whether the failure happens because of uncertainty or input changes. For example, in Figure 6, the objective is to insert a molar into the presented mandible. The inserted molar has small dimensions that may lead to several failure cases before a successful case. 

**Figure 7:**
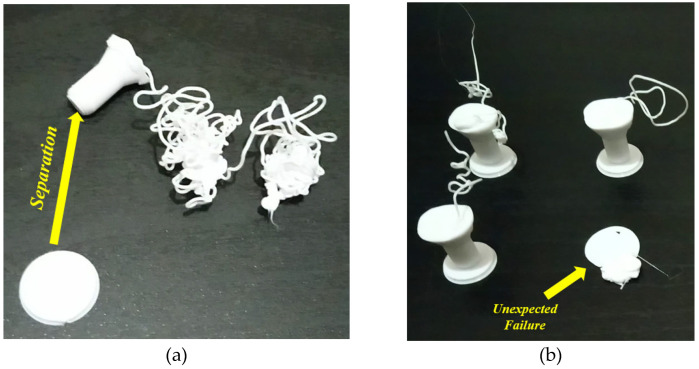
(**a**) Separation case, (**b**) unexpected failure case (images belong to 3d-printing-4u.com).

**Figure 8:**
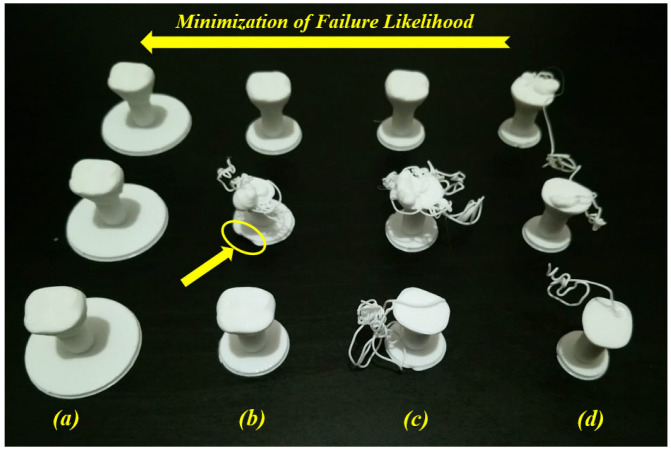
Minimization of failure likelihood for several raft margin values: (**a**) 10 mm, (**b**) 5 mm, (**c**) 4 mm, and (**d**) 3 mm (images belong to 3d-printing-4u.com).

As shown in Figure 7a, the raft margin value was 3 mm, and the failure occurred due to a separation of the printed part from its raft. The separation may have occurred due to print and/or travel speeds. To solve this problem, we reduced these (print and travel) speed values to half of their default values, and the position on the build platform was also changed. Although the separation problem was not repeated, the failure occurred several times, as shown in Figure 7b. In this figure, four failure cases occurred under the same input conditions (speed, raft, infill density and style); however, one of these failure cases occurred at the beginning of the printing process (unexpected failure). The cause of this failure is not known and thus represents an uncertainty case. Apart from the unexpected failure case, a required change in the input parameters should be carried out to obtain successful cases. So, the raft margin was increased to solve this problem. Figure 8a shows the best selection, where the margin was 10 mm, considering the same reduced speeds and the same position.

When testing the interval between 10 mm and 3 mm and for the same reduced speeds (print and travel), Figure 8b,c show failure and success cases for the same conditions where raft margin values are respectively 5 mm and 4 mm. These two condition cases represent uncertainty cases. In Figure 8b, a failure case occurred between two success cases for the same input conditions (same raft margin value (5 mm) and same reduced speeds). Here, we can detect that there is a problem at the first layer of the raft on the second printed part (see the yellow ellipse). The failure may have occurred due to the material extrusion at the beginning of the raft printing. However, in Figure 8c, the success occurred at the third printed part without detecting the failure cause (unknown cause). This kind of failure case due to unknown causes (Figure 7b and Figure 8c) needs to be treated in order to reduce the failure rates. For this example, in order to minimize the likelihood of failure, it is recommended to increase the raft margin values. Furthermore, after two success cases (raft margin was 10 mm) in Figure 8a, the print and travel speeds were doubled (back to default values) and another success case occurred. So, it can be concluded that the effect of the raft for these small parts is much higher than the effect of speeds. According to the uncertainty diagram presented in our previous work [19], uncertainty cases for AM-PLA using the FFF technique appear at two levels (design and process levels). In addition to the correlations between several parameters in this diagram, there is a correlation between the geometry (raft margin values) and the printing speed when dealing with this kind of dental application (complex geometries). It is then recommended to test several parameters to decrease the uncertainty effect, which leads to much more reliable AM processes, especially when dealing with dental applications with small dimensions (reduction of time, materials, and costs).

## 8. AM-PLA Using FFF for Dentistry with Consideration for Artificial Intelligence

**Figure 9:**
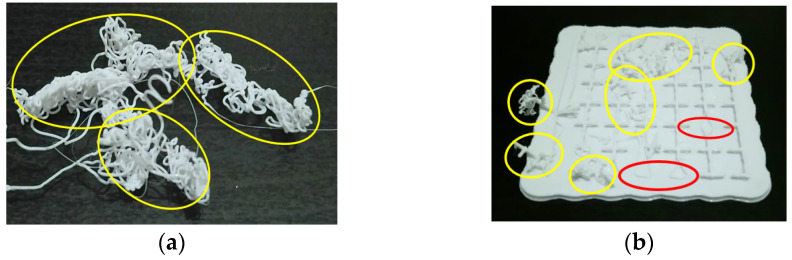
Two different failure modes: (**a**) Only Mode 1 in yellow ellipses and (**b**) Mode 1 in yellow ellipses and Mode 2 in red ellipses (images belong to 3d-printing-4u.com).

In order to establish international standards for AM-PLA using FFF, advanced technologies based on machine learning (ML) are required in order to deal with different types of data (numerical values, images, texts, etc.). According to the study requirements and the existing data types, several types of ML techniques have been reported in the literature [88,89,90,91,92]. For example, in AM processes, when training a model to diagnose a failure case from imaging data, each failure case must be labeled. Here, we can obtain a binary label (failure/no failure) for each failure case. This is called a supervised ML technique, and the supervision comes in the form of the provided labels. The ML task is then to predict the label of new failure cases based only on the given data features. On the other hand, it is possible to not have any labels, or to not even know what task we need to carry out with this data. We just need to examine the data and learn something about the structure of the data and the relationships between the different failure cases. In such cases, we can use an unsupervised ML technique. Conversely, according to the type of data, we obtain new classifications. Here, simple ML (or numeric ML) is essentially utilized in order to treat numeric data such as tables. Deep machine learning (DML) (or deep learning) [93] is mainly utilized in order to deal with images. In our previous work [19], a diagram to implement the DML technique, with the objective of treating several failure images in AM, was established considering the failure images for a single failure mode. Other modes can largely affect the AM process, even the machine elements. Figure 9 shows two different failure modes that need to be identified in two different learning ways (yellow and red ellipses). The image in Figure 9a can be divided into small squares (pixels) in order to test the light density, while the image in Figure 9b needs more advanced techniques to identify the failure. Two different consequences can occur: The first one is that the PLA material continues to flow from the extruder (Mode 1), while in the second one, the small separate material parts may enter the sliding guides of the printer (Mode 2), which may lead to wear and damage the machine. 

**Figure 10:**
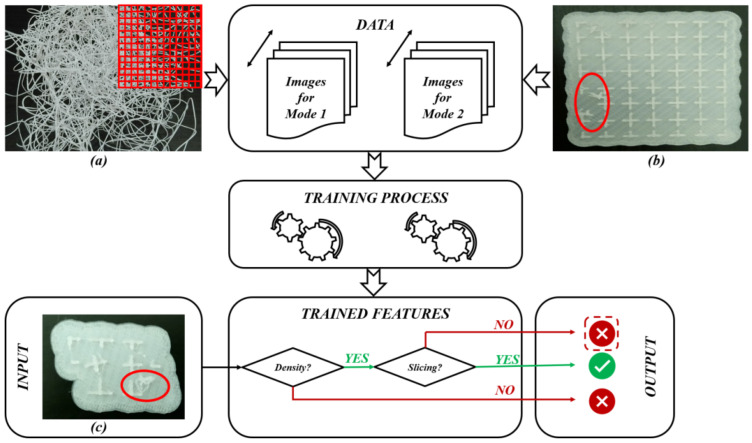
DML diagram for AM-PLA using FFF technique for dental applications: (**a**) Failure case for Mode 1, (**b**) Failure case for Mode 2 and (**c**) Test case (images (**a**–**c**) belong to 3d-printing-4u.com).

As shown in Figure 10, for the first failure mode (Mode 1), each image (such as image (a)) is divided into small parts to register information (density) about each part. When increasing the number of images (data), the resulting trained model will be improved. In DML, the features for each failure mode are learned as a result of the training process (learning process). For the second failure mode (Mode 2), a comparison with the slicing model can be carried out in order to identify the deviation levels. As illustrated in Figure 10, when introducing a new image (image (c)) as input to test, neural networks (several types of layers [93,94,95] are used to make a prediction to identify what the failure mode is). So, for the input image (c), the first test is to identify the density. When detecting a problem, it is recognized as a failure case (Mode 1). If the density situation is safe, a slicing test is next carried out to compare it to the original slicing file. When detecting any deviation, it should be recognized as a failure case (Mode 2). The test in Figure 10 for image (c) shows a failure case that belongs to the second failure mode (Mode 2). So, the goal of this DML strategy is to carry out end-to-end learning (feature extraction and classification).

Finally, we must mention that reinforcement machine learning (RML) (or reinforcement learning) [96,97,98] can be utilized at an advanced development stage to make a decision in each printing situation (for example, to continue, stop the AM process or modify process parameters). Without these surveillance and reactivity tasks, it may not only concern the waste of time and materials, but there may be other consequences affecting the performance of machine elements. For example, the platform may be removed from its glide tracks and other machine elements (glide tracks, door, filament tube and/or its clamp, etc.) can be damaged. So, there is a strong need to develop new strategies that allow one to integrate these ML technologies, which can be considered as core components of artificial intelligence (AI) [99,100,101,102], in order to pave the way to extend AM-PLA using FFF to more applications in dentistry since, in addition to their simple implementation, the PLA material itself and the applied FFF technique have several advantages in AM areas. 

## 9. Challenges, Issues, and Future Perspectives

**Figure 11:**
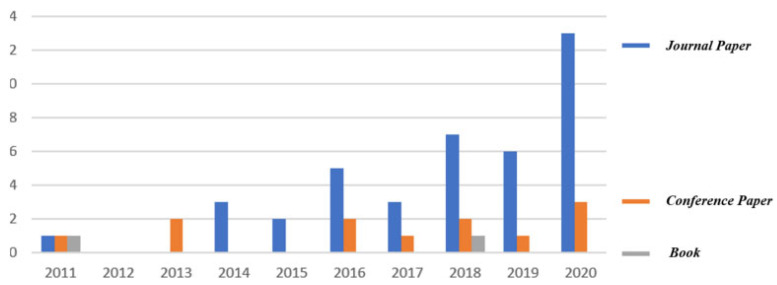
Trend analysis of the studied topic according to Lens’s website (www.lens.org).

During the last three decades, a simple trend analysis according to Lens’s website (www.lens.org, accessed on 27 April 2023), shows that the number of different publications on this topic totaled only 56 (two books, 12 book chapters, one conference proceeding paper, one dissertation and 40 journal articles) as shown in Figure 11. 

The first journal article was published in 2011, while the real start of this topic began a few years ago (46 publications from 2016). In order to guarantee the continuity of this topic, the different issues and challenges related to it should be first identified, and some future perspectives should be next suggested considering several development axes (lattice structures, support structures, etc.) of this topic.

**Figure 12:**
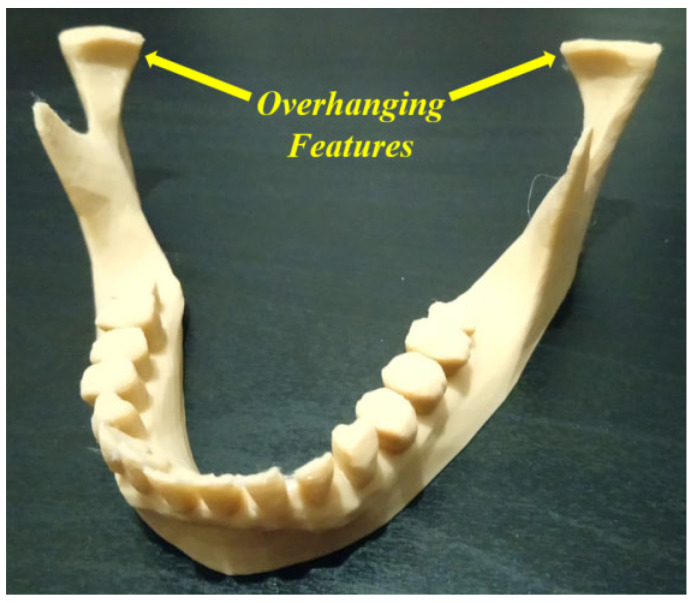
Overhanging features (image belongs to 3d-printing-4u.com).

The main issue is related to the identification of uncertainties (for example, failure causes and consequences) with the objective of reducing the rates of different failure modes. For example, in Figure 8, we proposed increasing the raft margin values for this specific case in order to minimize the failure likelihood. Without solving this kind of problem, a waste of time and materials can occur, and the quality of the additively manufactured products can be affected. Dental applications are generally related to complex geometries, which leads to the appearance of additional uncertainty cases (failure modes) during the AM processes. Another example of these complexities can be represented by the existence of overhanging features, which lead to extruding materials in the air. According to gravity laws, suitable support structure types need to be added at the slicing stage to prevent a failure occurrence [82,103,104,105]. As the support structures are obligatory to continue the AM process, they increase costs and require additional finishing processes. At the end of the AM process, these supports must be removed carefully by using appropriate tools such as needles, pliers, knives, cutters, etc. The selection of these tools should be carried out considering the shape and material of the fabricated components. To decrease these complexities and costs, it is very important to simulate the AM process at the slicing stage in order to find a suitable way to avoid or reduce the support structure requirements. For example, in Figure 12, the illustrated mandible condyles generally require support structures; however, when changing certain parameters and simulating the AM process on the slicing software, it is possible to avoid using these support structures, which saves costs and provides printed parts with a good quality.

The challenges can be represented by cost reduction, reduction in material consumption, and quality improvement. Furthermore, it is very important to take other criteria such as sustainability into account during the different developments. Therefore, there is a strong need to increase the efforts toward developing advanced techniques to overcome the different challenges in this complicated topic, which combines several research axes (complex geometries in dental applications and AM-PLA using FFF technique). Previously, optimization techniques were used to reduce costs, waste of materials, time, etc.; however, currently, AI and automation are also helpful techniques in order to overcome these challenges. 

It is true that AM-PLA by FFF is used for educational models, tooling models, visual aids, and alpha prototypes [15,106], but when changing certain parameters, we improve certain mechanical properties of the printed PLA components to extend their uses to additional applications. For example, according to Amirruddin et al. [107], with increases in layer thickness, frictional force, and other parameters, a good improvement was shown in wear resistance, which can be applied in certain dentistry applications. Additional developments of the mechanical properties of this sustainable material with several processing benefits and tasks can be found in the literature [15,108]. 

**Figure 13:**
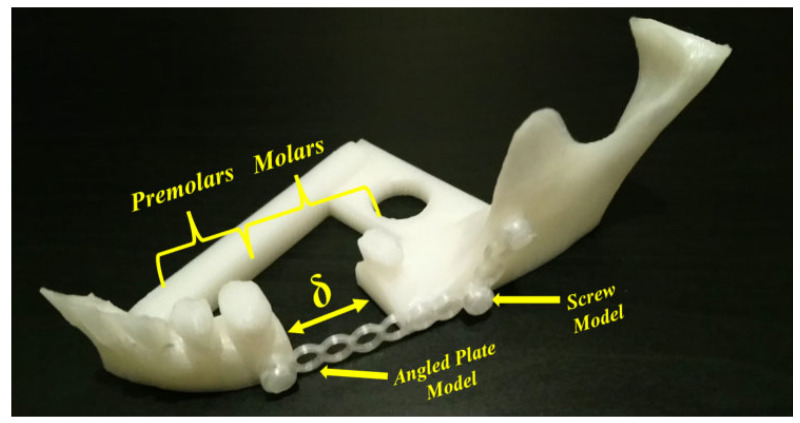
Fractured mandible model (image belongs to 3d-printing-4u.com).

In addition, according to Myers et al. [38], cellular structure applications in medicine are developed for orthopedic scaffolds due to their low elastic modulus values, high compressive strength, etc.; however, their applications can be extended to cover surgical operations in dentistry (maxilla and mandible). Figure 13 shows a special fracture with partial bone loss in the mandible body part. The operation can be carried out by using long angled plates. Here, we have a body fracture with partial bone loss at the first and second molar levels. For this kind of fracture, an angled plate with screws can be used for fixation and the patient will be left with a bony defect with a possibility of later repair. The numbers of screws and the length of the used angled plate in Figure 13 represent the main idea of fixation; however, the detailed representation to bridge the bone defect depends on the patient’s clinical case. For more details, the interested reader can refer to [109,110,111,112].

**Figure 14:**
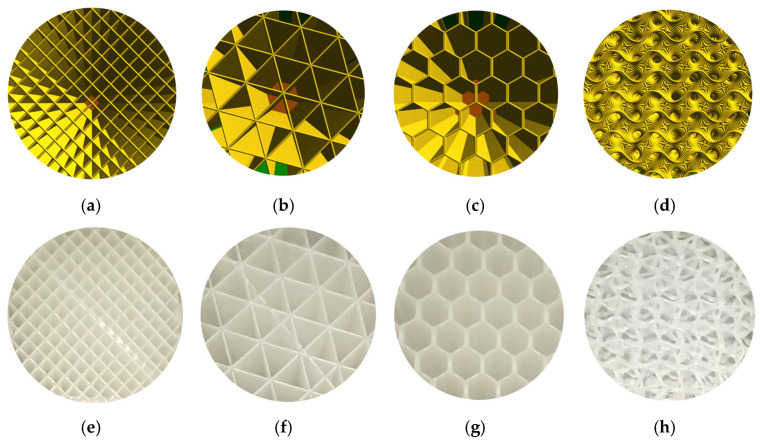
Infill styles for slicing models: (**a**) linear, (**b**) triangular, (**c**) hexagonal, and (**d**) wavy forms; and for AM-PLA models: (**e**) linear, (**f**) triangular, (**g**) hexagonal, and (**h**) wavy forms (images belong to 3d-printing-4u.com).

A cellular PLA structure can be used between the fracture’s surfaces to replace the lost bone part. During the healing period, the cellular PLA structure can guide the direction of the spongy (trabecular) bone ingrowth. Because of the PLA biodegradability, the spongy bone can replace the fixed cellular PLA structure. For example, when slicing a geometry, in addition to the existing models (Schwarz Primitive, Schoen Gyroid, etc.), there are several infill styles such as linear, triangular, hexagonal, and wavy styles (Figure 14a–d) that can be provided in the slicing software (ex. FLASHPRINT). Additionally, the corresponding AM-PLA sections shown in Figure 14e–h are executed using Adventurer 3+. The logic of osteointegration leads one to consider that the wavy style (Figure 14d,h) seems to be the best solution for this kind of surgical operation. 

However, it is possible to create our own models by experimentation (trials and tests) or by using topology optimization technology. Regarding the use of the FFF technique, there is still a big challenge to use it as a prototyping technique since the final printed components have several problems concerning the mechanical properties, especially anisotropy and tensile strength. Several developments are needed in order to improve the mechanical properties of the printed parts; however, AM-PLA using the FFF technique allows us to manufacture the resulting optimal topology in a simple and quick way [113]. When performing topology optimization, we suggest here to add new constraints to the optimization problem to reduce the effect of anisotropy errors. In addition, other challenges can be found when changing the initial design space several times, as various resulting topologies can be obtained. To control this problem, reliability analysis can be integrated into the topology optimization process. In this way, sensitivity analysis plays an important role in determining the most effective parameters in the studied structure. A reliability-based topology optimization (RBTO) model can be used instead of deterministic topology optimization (DTO) [114]. So, to formulate the topology optimization problem in Figure 13, we define the initial domain (providing the optimized and non-optimized areas) and also the boundary conditions and material properties. The boundary conditions (loads and displacements) can be determined clinically [115,116]. So, the left space (δ) in Figure 13 can be subjected to topology optimization technology to find the best topology considering several constraints such as mechanical, medical, and other limitations. The resulting cellular PLA structure should help to accelerate the healing process. In order to obtain the best topology, several improvements can be made. For example, when detecting new failure modes, new constraints can be added to the topology optimization formulations, especially when dealing with composite PLA materials. Furthermore, the diagram in Figure 10 can be improved when detecting new failure modes to be more generalized. 

**Figure 15:**
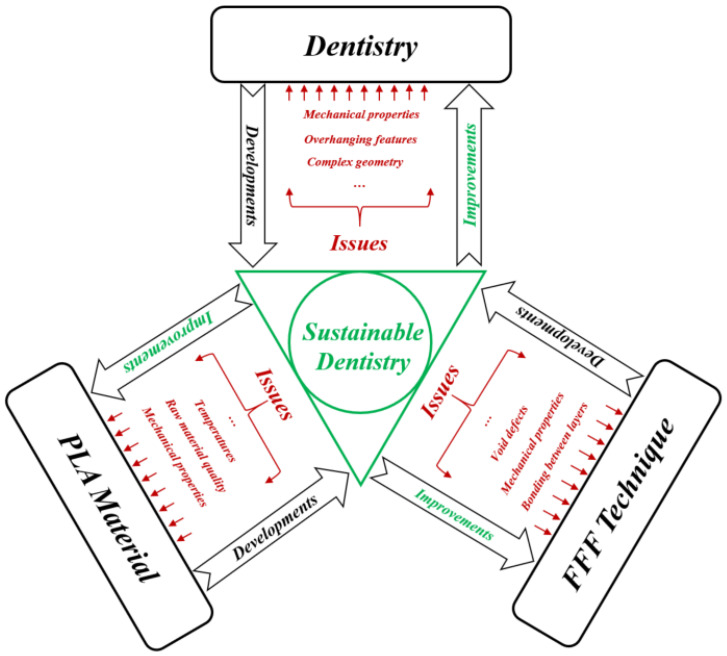
Sustainable dentistry diagram.

This review provides the reader with several suggestions for developing this topic at the design and manufacturing stages in order to pave the way to sustainable dentistry. Figure 15 provides a diagram summarizing the road to develop tools that solve the existing issues in the three axes (dentistry, PLA material and FFF technique) with the objective of achieving the sustainable dentistry level. These developments will also lead to several future improvements that can be useful to other fields, especially from a sustainability standpoint. For example, when improving mechanical properties such as roughness and hardness of the additively manufactured products, the PLA material and FFF technique will cover other dental applications. In addition to dentistry, the use of the developments in PLA material and the FFF technique will be expanded to many applications in several other fields. 

## 10. Conclusions

The objective of the current review was to provide roadmaps and innovative ideas to researchers in order to develop novel strategies for industrializing the AM technology, especially when dealing with AM-PLA material using the FFF technique in dentistry. Reducing the cost and increasing productivity are the main challenges to the industrialization of AM technology. In this review, the AM-PLA material was selected due to its many advantageous properties such as biocompatibility, biodegradability, and sustainability. The FFF technique was also selected due to its simplicity, common usability, and cost-effective maintainability. However, due to the geometrical complexity, using AM-PLA material with the FFF technique in dentistry is a major challenge. Several new ideas were then proposed to solve the different issues presented in this review, such as support structures and cellular structures. In addition, when using the FFF technique to fabricate PLA material, we currently provide prototypes where several research axes are needed to study the resulting dental products from a resistance standpoint. One of these axes can focus on the development of new composite PLA materials with the objective of improving the mechanical properties, allowing us to cover other dental applications. In future works, our objective will be to focus on maintainability and AI technology in order to form a complete loop for the life cycle of AM products and move closer to a sustainable world.

## Figures and Tables

**Table 1:** Recent works in additively manufactured materials for dental applications.

Authors	Material	AM Technique	Dental Application	Results
Bae et al. [43]	3Y-TZP ceramics	SLM + CIP	Dental crown, prostheses, restoration.	Foundation of SLS/CIP technology for 3Y-TZP dental ceramics
Muta et al. [44]	PVA	FDM	Provisional dental crown	Good accuracy
Arnesano et al. [45]	Alumina-Ceramic	FDM	Dental crown	Energy efficiency
Revilla-León et al. [46]	Co-Cr alloy	SLM + CM	Dental prostheses	Improved roughness with SLM process
Baciu et al. [47]	Co–Cr–W alloy	SLM	Dental inlays and bridges	Increased hardness

## Abbreviations

ABS	Acrylonitrile butadiene styrene
AI	Artificial Intelligence
AM	Additive Manufacturing
AM-PLA	Additively Manufactured Polylactic acid
CIP	Cold Isostatic Pressing
DML	Deep Machine Learning
DMLS	Direct Metal Laser Sintering
DTO	Deterministic Topology Optimization
EBM	Electron Beam Melting
FDM	Fused Deposition Modeling
FFF	Fused Filament Fabrication
LOM	Laminated Object Manufacturing
ML	Machine Learning
PC	Polycarbonate
PCL	Polycaprolactone
PEI	Polyetherimide
PLA	Polylactic acid
PVA	Polyvinyl alcohol
RBTO	Reliability-Based Topology Optimization
RML	Reinforcement Machine Learning
SLA	Stereolithography
SLM	Selective Laser Melting
SLS	Selective Laser Sintering
